# CD271 Identifies a Subpopulation with Enhanced Neural-like Potential Within Wharton Jelly Derived Mesenchymal Stem/Stromal Cells

**DOI:** 10.3390/ijms27114896

**Published:** 2026-05-28

**Authors:** Agnieszka Smolinska, Magdalena Chodkowska-Michalowska, Klaudia Radoszkiewicz, Aleksandra Bzinkowska, Anna Sarnowska

**Affiliations:** Translational Platform for Regenerative Medicine, Mossakowski Medical Research Institute, Polish Academy of Sciences, 02-106 Warsaw, Poland

**Keywords:** mesenchymal stem/stromal cells, CD271, FACS, neuronal differentiation, neural crest derived stem cells, neural crest

## Abstract

The heterogenous mesenchymal stem/stromal cells (MSCs) express the surface antigens associated with distinct cell subpopulations. CD271, characteristic of stem cells derived from the neural crest, could indicate cells with a unique phenotype. The study examined whether the CD271+ subpopulation characterized by better stem and neural properties than the heterogeneous MSC population. The initial Wharton jelly-derived MSCs (WJ-MSCs) population was divided into two subpopulation: CD271-positive (WJ-MSC-CD271+) and CD271-negative (WJ-MSC-CD271−) with Fluorescence-Activated Cell Sorting (FACS). We compared the clonogenic potential and neural marker expression under standard culture conditions and in the presence of nerve tissue components—cerebrospinal fluid (CSF) or nerve tissue fragments (hippocampus). FACS allowed the enrichment of CD271+ cells from 1% to approximately 50%. WJ-MSC-CD271+ is characterized by significantly more self-renewal cells and increased expression of neuronal genes than WJ-MSC-CD271−. Under co-culture with CSF or hippocampal fragments, WJ-MSC-CD271+ contained more cells expressing Β-III-tubulin as well. Finally, we reported that stimulation with epithelial growth factor (EGF) and basal fibroblast growth factor (bFGF) enhanced CD271+ numbers in the initial population and stabilized them in further cell culture. WJ-MSC-CD271+ cells showed improved potential for differentiation into neural progenitors, although further research is needed for their potential use in neurological diseases.

## 1. Introduction

Despite many years of studies, numerous approaches, and available tools, the treatment of nervous system disorders and injuries remains problematic. Stem cells (SCs) are listed among the proposed solutions, with a wide range of sources and general concepts of how to isolate, cultivate, and inject them into the patient. Neural stem cells (NSCs) and neural progenitor cells (NPCs) appear to be the most intuitive option, but their isolation feasibility is limited when it comes to the derivation of human primary cells [1,2]. Induced pluripotent stem cells (iPSCs) and embryonic stem cells (ESCs) are considered due to their broad differentiation potential, but their choice also presents some considerable limitations [2,3,4]. Mesenchymal stem/stromal cells (MSCs) are currently the most frequently chosen type of SCs for nervous tissue treatment in the ongoing clinical trials [4]. Despite their multipotency, many research groups proposed protocols for receiving neuron-like cells from the MSC population [5], but the received cells did not always exhibit the functional features of neurons. Indeed, MSC engraftments did achieve therapeutic improvements in animal models, but rather due to their paracrine activities, neuroprotection, and inflammation reduction than to differentiation and integration [6,7].

Although MSCs are widely considered to exert their therapeutic effects primarily through paracrine mechanisms rather than through direct differentiation, numerous studies, including our previous works, reported the expression of neural markers together with an electrophysiological activity following in vitro differentiation [8]. The observed discrepancies raise a question for possible interpretation, while the answer could lie in the intrinsic heterogeneity of the MSC population containing cells with distinct developmental origins. Among them, cells derived from the neural crest (NC) can be found [9,10,11]. Neural crest-derived stem cells (NCSCs), even isolated from adult tissues, still possess their neuroectodermal characteristics [12,13,14], which could explain the spontaneous expression of neural markers by MSCs [6,15]. CD271—a surface antigen associated with NC—was identified in MSC tissues, such as bone marrow and adipose tissue [11,16,17,18], supporting this speculation.

Researchers propose a variety of CD271 roles within MSCs: stemness [16,19,20], immune response [21], osteogenesis [22,23], bone or cartilage regeneration [23,24] and angiogenesis [25,26]. Despite CD271 association with NCSCs, direct evidence supporting neural differentiation-related properties of MSC-CD271+ cells remains largely unknown. The majority of available studies investigating CD271+ MSCs focused on adult sources such as bone marrow or adipose tissue and primarily characterized proliferation or mesenchymal differentiation properties in the undifferentiated state [16,19,20]. In contrast, Wharton’s jelly (WJ) represents a neonatal source of young MSCs with high expansion potential [27,28] and containing a small but stable CD271+ subpopulation [16]. Since MSC properties are strongly influenced by tissue source, isolation strategy, and donor age, neonatal CD271+ cells may represent a biologically distinct population.

**Figure 1:**
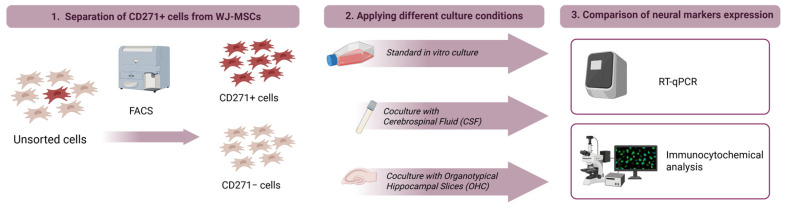
Overview of experimental steps (Created in BioRender. Kamińska, A. (2026) https://Bio-Render.com/7nuyv3m) (Accessed on 22 May 2026).

The main goal of the study was to evaluate whether CD271+ cells from the WJ-MSC population are capable of neural-like differentiation under conditions more closely resembling the physiological neural niche. Instead of using classical neuronal induction media, we intentionally applied experimental conditions based on complex neural tissue-derived signaling environments, which better mimic the in vivo neural microenvironment. CD271+ subpopulation was separated with fluorescence-Activated Cell Sorting (FACS), resulting in the generation of two populations: negative (WJ-MSC-CD271−) and positive (WJ-MSC-CD271+) (Figure 1). In the first experimental stage, analyzed populations were characterized in the context of self-renewal potential and the spontaneous expression of neuronal and glial markers. We also examined the dynamics of CD271+ cells in further WJ-MSC in vitro culture for the next two passages after FACS. In the next stage, positive and negative subpopulations were cultured with nervous tissue components—cerebrospinal fluid (CSF) or fragments of native tissue as an organotypic hippocampal slice culture (OHC). After the co-culture, examination of the mentioned markers’ expression was repeated. In this manner, we aimed to identify whether a specific subpopulation within the heterogeneous WJ-MSC population possesses an intrinsic predisposition to spontaneously respond to neural tissue-derived environmental cues. We hope to clarify whether the presence of the CD271 antigen could predict a better cell differentiation capability and whether a selected MSC subpopulation could serve as a pool of neural progenitor cells for nervous system therapies.

## 2. Results

### 2.1. Characteristics of WJ-MSCs

**Figure 2:**
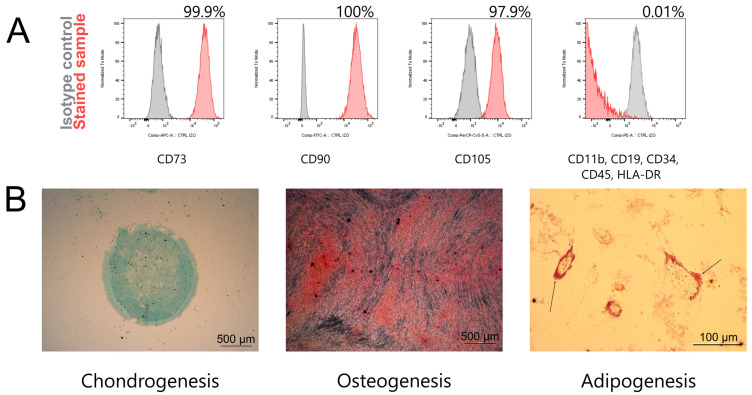
The characteristics of Wharton jelly-derived mesenchymal stem/stromal cells (WJ-MSCs) are presented in accordance with the recommendations of the International Society for Cell and Gene Therapy (ISCT). (**A**). Analysis of surface antigens: CD73, CD90, CD105 and negative mix (CD11b, CD19, CD34, CD45 and HLA-DR); flow cytometry—representative histograms. (**B**). Multipotent differentiation of WJ-MSCs: chondrogenesis, osteogenesis and adipogenesis. Black arrows indicate lipid droplets in the cell, stained with oil red O.

Before the start of the experiments, the criteria suggested by the International Society for Cell and Gene Therapy (ISCT) were examined for the initial population. WJ-MSCs cultured under standard conditions exhibited a typical spindle-shaped morphology and expressed the following surface markers: CD73, CD90 and CD105, and lacked expression of CD11b, CD19, CD34, CD45 and HLA-DR (Figure 2A). WJ-MSCs also differentiated into chondrocytes, osteocytes and adipocytes (see Figure 2B), as confirmed by alcian blue, alizarin red and oil red O staining, respectively. These observations are consistent with the ISCT definition and confirm the characteristics of the MSCs.

### 2.2. Sorting Efficiency and CD271+ Population Maintenance

**Figure 3:**
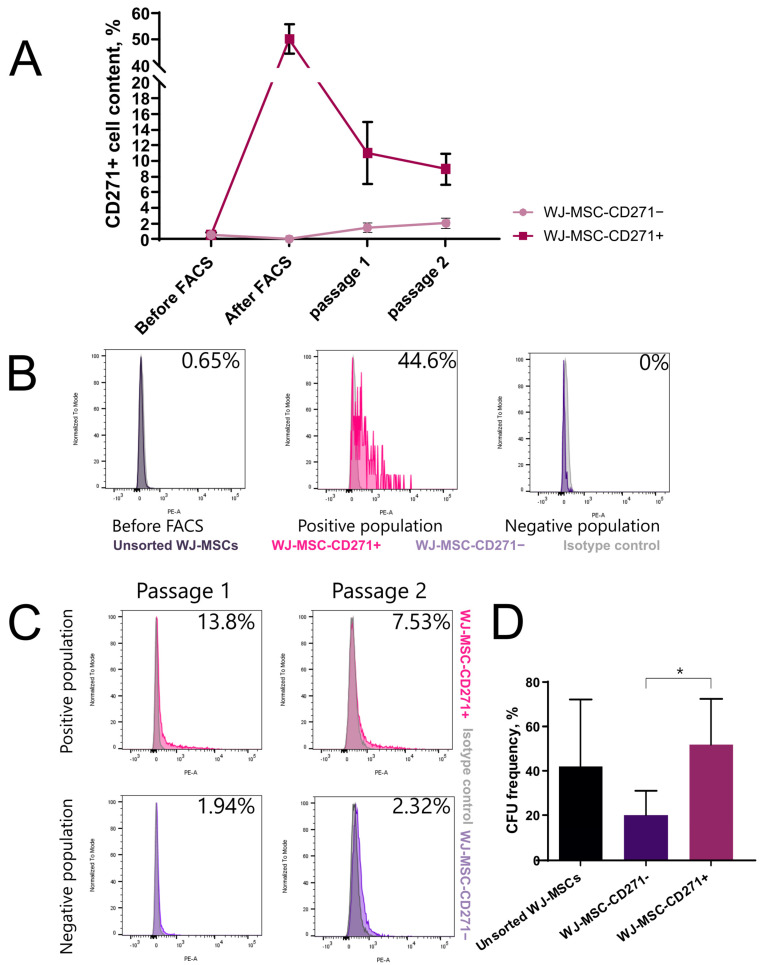
Fluorescence-Activated Cell Sorting (FACS) results and colony-forming unit (CFU) frequency assay. (**A**). CD271+ cell content dynamics before FACS, directly after FACS and for 2 passages post FACS for positive (WJ-MSC-CD271+) and negative (WJ-MSC-CD271−) populations. (**B**). CD271+ cell content before FACS and directly after FACS in positive and negative populations; flow cytometry—representative histograms. Isotype control—grey histograms. (**C**). CD271+ cell content during further in vitro culture after FACS for positive and negative populations; representative histograms. (**D**). CFU frequency assay for initial (unsorted WJ-MSCs), negative and positive populations. Results are presented as mean values of at least 3 experiments ± SD; *p*-value for * < 0.05.

The initial population of WJ-MSCs exhibited a small subset of CD271+ cells, accounting for less than 1% of the total population (Figure 3A,B). After FACS, the following subpopulations were obtained: a positive population (WJ-MSC-CD271+) containing approximately 50% CD271+ cells and a negative population (WJ-MSC-CD271−) containing less than 0.5% CD271+ cells (see Figure 3A,B). All three populations (the initial population, the positive subpopulation and the negative subpopulation) were seeded and cultured for the next two passages. CD271 expression gradually decreased in the WJ-MSC-CD271+ subpopulation, which had around 10.9% CD271+ cells in the first passage after FACS and 8.9% in the second passage. In contrast, we observed a slight increase in CD271 expression in the WJ-MSC-CD271− population (1.5% in the first passage and 2% in the second passage) (Figure 3A,C).

Due to the small number of CD271+ cells in the initial population, some technical issues were encountered (Table 1). The numbers collected in the positive population were low compared to the initial population, with CD271+ cell recovery below 25%. The CD271+ population was enriched 110-fold during FACS, but the general efficiency was around 18%. Cell viability before and after sorting was similar, so FACS and transportation did not affect the cells.

The colony-forming unit (CFU) assay confirmed the lower clonogenic potential of the CD271 cells. There were no significant differences in CFU frequency between the initial and positive populations (42.2% ± 29.9 and 52% ± 20.4, respectively). However, the negative population formed significantly fewer colonies (20% ± 11.2). Our observations indicate the stem/progenitor characteristics of CD271+ cells within the heterogeneous WJ-MSC population (Figure 3D).

### 2.3. Expression of Neural Markers Under Standard Culture Conditions

**Figure 4:**
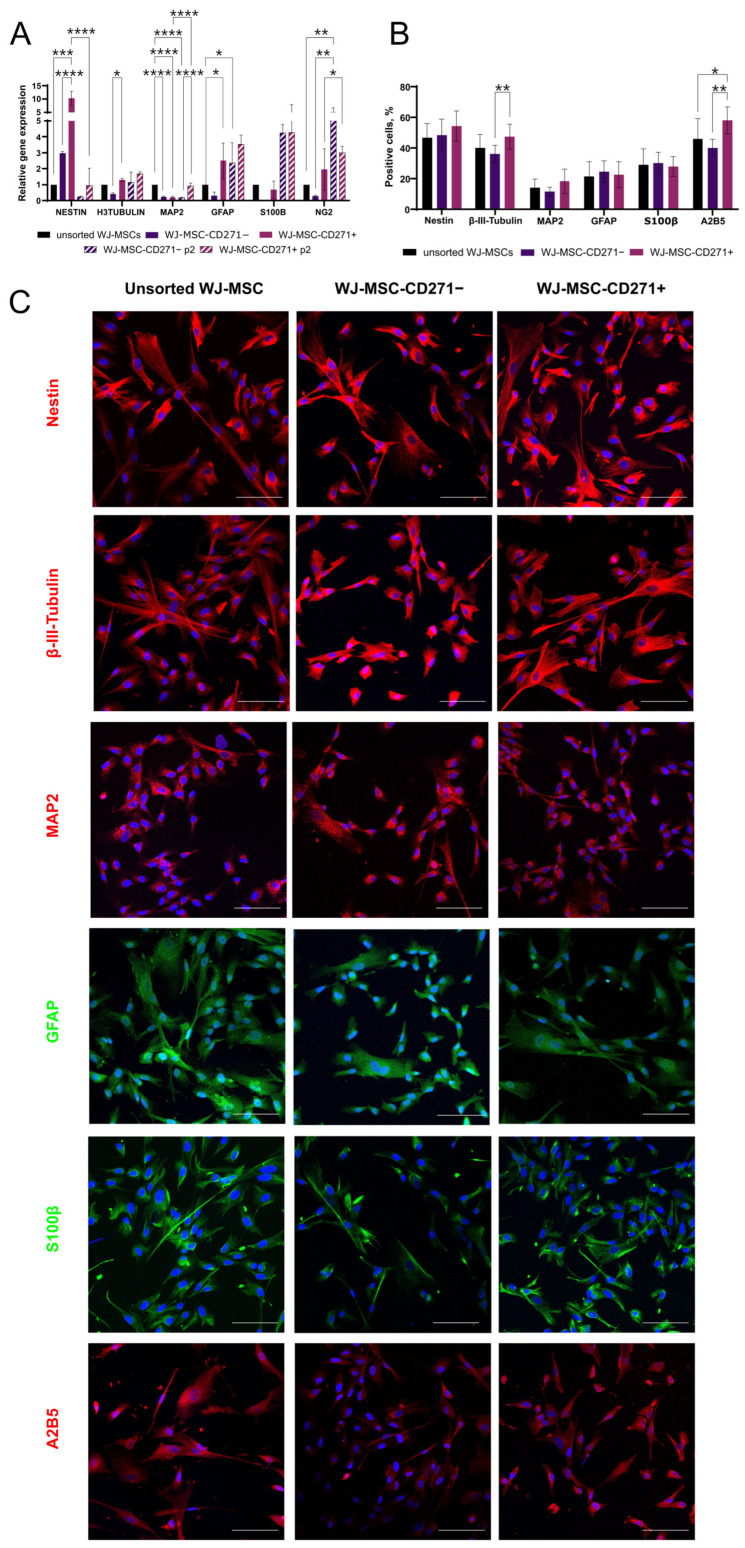
Neural markers expression in WJ-MSCs populations. Following groups were compared: unsorted WJ-MSCs, CD271-negative population (WJ-MSC-CD271−) and CD271-positive population (WJ-MSC-CD271+). (**A**). Relative gene expression level (fold change, mean ± SD) of genes associated with the neural tissue. Quantitation was determined relative to GAPDH by quantitative real-time PCR. Changes in gene expression are shown relative to the unsorted WJ-MSC population (value = 1). The expression was analyzed for populations cultured in vitro for 1 and 2 passages (p2). Results shown are the mean of 3 independent RNA isolations, *p*-value for * < 0.05, ** < 0.01, *** < 0.001, **** < 0.0001. (**B**). Immunocytochemical analysis of cells positive for the specific neural markers. Results shown are as a mean of 3 experiments, *p*-value for * < 0.05, ** < 0.01. (**C**). Immunocytochemical stainings for the analyzed neural markers. Scale bars = 100 µm.

The RNA expression and protein levels of specific neural markers were determined for the initial, positive and negative populations (see Figure 4). To this end, we analyzed markers associated with neural progenitors (Nestin), neurons (β-III-tubulin and MAP2), glial cells (GFAP and S100β) and oligodendrocyte precursors (NG2 and A2B5).

Gene expression analysis revealed the unique gene profile of WJ-MSC-CD271+ cells (see Figure 4A). Following the first passage after FACS, CD271+ cells exhibited increased NESTIN expression. In the second passage, NESTIN expression decreased while H3TUBULIN and MAP2 gene expression increased in WJ-MSC-CD271+. CD271+ cells exhibited higher GFAP and NG2 expression directly after FACS; however, in the next passage, GFAP and NG2 expression increased in CD271− cells, reaching levels equal to or higher than those observed in CD271+ cells. No significant differences in S100B expression were reported between groups; however, its expression tended to increase with further culture.

As gene expression alone was insufficient for assessing the neural identity of CD271+ cells, immunofluorescence staining was performed to visualize the analyzed markers (Figure 4B,C). Immunocytochemical analysis revealed a significant increase in β-III-tubulin and A2B5 expression in WJ-MSC-CD271+ cells, reflecting the observed tendencies in the relative gene expression analysis.

### 2.4. Influence of Nervous Tissue Components on CD271+ Population Neural Phenotype

**Figure 5:**
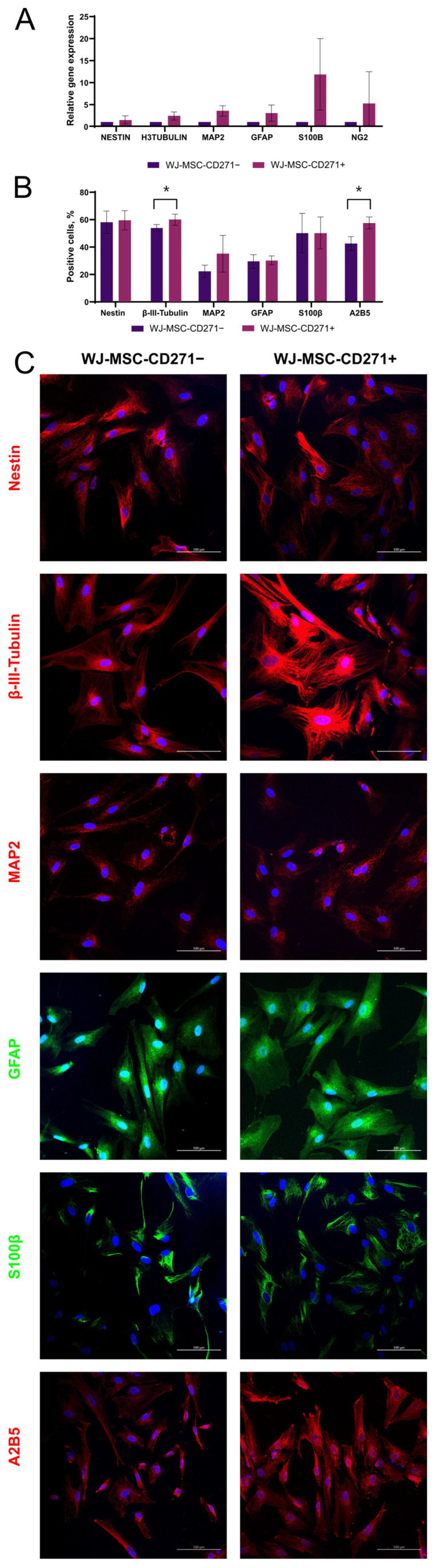
Neural markers expression of WJ-MSCs populations after coculture with Cerebrospinal fluid (CSF). Following groups were compared: CD271-negative population (WJ-MSC-CD271−) and CD271-positive population (WJ-MSC-CD271+). (**A**). Relative gene expression level (fold change, mean ± SD) of genes associated with neural tissue after coculture with CSF. Quantitation was determined relative to GAPDH by quantitative real-time PCR. Changes in gene expression are shown relative to WJ-MSC-CD271− (value = 1). Results shown are a mean of 3 independent RNA isolations. No significant differences were observed between groups. (**B**). Immunocytochemical analysis of cells positive for the specific neural markers after culture with CSF. Results shown are a mean of 3 experiments, *p*-value for * < 0.05. (**C**). Immunocytochemical stainings for the analyzed neural markers. Scale bars = 100 µm.

**Figure 6:**
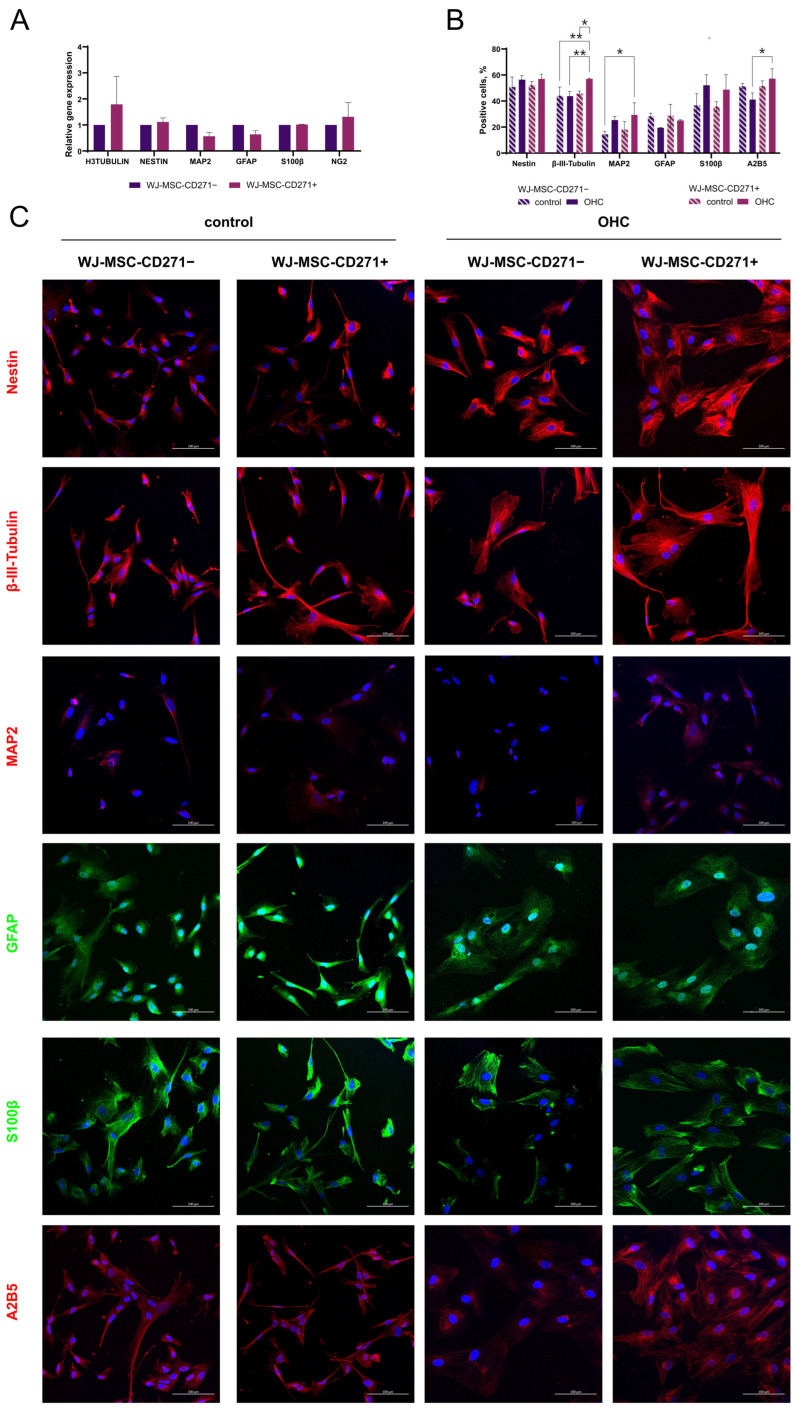
Neural marker expression of WJ-MSCs populations upon organotypic hippocampal slice cultures (OHC). Following groups were compared: CD271-negative population (WJ-MSC-CD271−) and CD271-positive population (WJ-MSC-CD271+) that were cultured in standard culture conditions, without hippocampal slices (control) or with hippocampal slices (OHC). (**A**). Relative gene expression level (fold change, mean ± SD) of genes associated with neural tissue after culture with OHC. Quantitation was determined relative to GAPDH by quantitative real-time PCR. Changes in gene expression are shown relative to WJ-MSC-CD271− (value = 1). Results shown are a mean of 3 independent RNA isolations. No significant differences were observed between groups. (**B**). Immunocytochemical analysis of cells positive for the specific neural markers after culture with OHC. Results shown are the mean of 3 experiments ± SD, *p*-value for * < 0.05, ** < 0.01. (**C**). Immunocytochemical stainings for the analyzed neural markers. Scale bars = 100 µm.

To assess the presence of a neural phenotype in a neural niche, cells from positive and negative subpopulations were cultured in CSF derived from healthy donors (Figure 5), or cocultured with OHC (Figure 6). After seven days, the expression of previously described markers was examined.

During CSF co-culture, we observed higher expression of all analyzed genes in CD271+ cells. However, due to the high standard deviation, the reported values were not statistically significant (Figure 5A). However, immunocytochemical analysis confirmed a higher content of β-III-Tubulin and A2B5 in the CD271+ population (Figure 5B,C).

Next, the cells were cultured in the presence or absence of OHC (as a control group). Gene expression analysis revealed no significant differences in gene expression between the populations cultured with OHC (Figure 6A). Immunocytochemical staining revealed that OHC-cultured WJ-MSC-CD271+ cells contained more β-III-tubulin- and A2B5-positive cells than OHC-cultured WJ-MSC-CD271− cells (Figure 6B,C), thus confirming an increased content of the two markers in WJ-MSC-CD271+ cells under all experimental conditions.

### 2.5. Effects of EGF and bFGF on CD271+ Expression in WJ-MSCs

In the final step, we examined the effect of two standard mitogens used in stem cell culture, epidermal growth factor (EGF) and basal fibroblast growth factor (bFGF), on the dynamics of the CD271 subpopulation. The initial WJ-MSC population was pre-treated with EGF and bFGF one passage before FACS. Stimulation continued for the next two passages after FACS for the resulting subpopulations. Our analysis included a variant where EGF and bFGF were withdrawn after FACS to determine whether the observed effect was permanent or transient.

**Figure 7:**
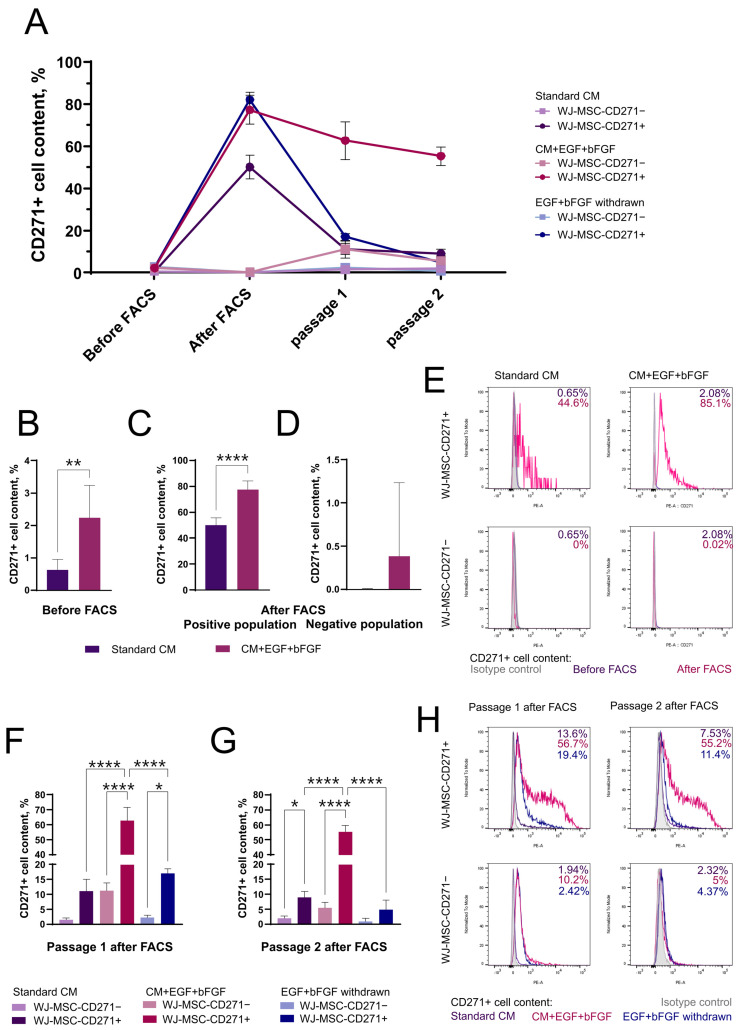
CD271 expression changes after stimulation of epidermal growth factor (EGF) and basal fibroblast growth factor (bFGF). Following conditions were compared: standard culture medium (standard CM), culture medium with EGF and bFGF used before and after FACS (CM + EGF + bFGF) and when EGF and bFGF were withdrawn after FACS (EGF + bFGF withdrawn). (**A**). CD271+ cell content dynamics during cell culture before and after FACS for positive (WJ-MSC-CD271+) and negative (WJ-MSC-CD271−) populations. (**B**–**D**). CD271+ cell content before FACS and after FACS for positive and negative populations. (**E**). Representative histograms for a flow cytometry analysis for CD271 expression for positive and negative populations before and after FACS. (**F**,**G**). CD271+ cell content for 2 passages post FACS sorting for positive and negative populations. (**H**). Representative histograms for a flow cytometry analysis for CD271 expression for positive and negative populations cultured after FACS. For (**B**–**D**) and (**F**,**G**) results are presented as mean values of at least 3 experiments ± SD; *p*-value for * < 0.05, ** < 0.01, **** < 0.0001.

EGF and bFGF were found to significantly increase CD271 expression in the initial WJ-MSC population by up to 2.2% (see Figure 7A,B), thereby improving FACS outcomes (see Supplementary Table S1). The number of CD271+ cells in the WJ-MSC-CD271+ population increased to around 77% with EGF and bFGF stimulation (Figure 7A,C,E), while the negative population still contained fewer than 0.5% CD271+ cells (Figure 7A,D,E). Further in vitro culture under EGF and bFGF stimulation resulted in an elevated number of CD271+ cells in the WJ-MSC-CD271+ population (above 55%) (Figure 7A,F,H). However, a slight increase in the number of CD271+ cells in the WJ-MSC-CD271− population was observed (1st passage: 11.1% ± 2.68; 2nd passage: 5.5% ± 1.82) (Figure 7A,F,H). However, these differences were not statistically significant compared to the values reported for the standard culture medium. EGF and bFGF increased the number of CD271+ cells in the analyzed populations, but withdrawal restored the population to its previously reported size. The CD271+ cell content of the WJ-MSC-CD271+ population decreased sharply during the first (17% ± 1.59) and second (9% ± 1.98) passages. The addition of EGF and bFGF improved FACS efficiency and increased the CD271+ pool in the WJ-MSC population, suggesting that these mitogens are essential for maintaining a progenitor cell pool in the MSC population.

## 3. Discussion

### 3.1. CD271 in the MSC Population—A Potential Role

Currently, mesenchymal stem/stromal cells (MSCs) are the most frequently chosen type of stem cell (SC) for treating nerve tissue in ongoing clinical trials for central nervous system (CNS) disorder treatment [4]. Although they are classified as multipotent, some studies have reported the spontaneous expression of proteins associated with neurons and glial cells by MSCs [6,15], postulating possible differentiation toward neurons [5,29]. While numerous studies reported neural-like differentiation of MSCs, in most cases these observations were limited to morphological changes or expression of selected neural markers. In contrast, only a very limited number of groups, including our group, demonstrated more advanced and functionally relevant neuronal features, such as electrophysiological activity and action potential generation [30,31,32,33]. The reasons for these inconsistencies remain unclear. One possible explanation is the intrinsic heterogeneity of MSC populations and the presence of only a small subset of cells possessing genuine neurogenic competence [18,34]. According to some studies, a small proportion of cells in the MSC population could originate from the neural crest, which appears during nervous system development [9,10,11,16,17].

During epithelial-to-mesenchymal transition, neural crest-derived stem cells (NCSCs) delaminate from the neural crest and migrate to different areas of the developing organism. These cells give rise to neurons and glial cells of the peripheral nervous system (PNS), neuroendocrine cells, melanocytes and the craniofacial skeleton [35]. NCSCs can be isolated from adult tissues and still differentiate efficiently towards neurons and glial cells [12,13]. The following genes and markers are used for NCSC identification: HNK1, SLUG, SNAIL, FOXD3, TWIST, SOX10, Nestin and CD271 [9,36,37,38,39]. Here, we focus on a population exhibiting the CD271 surface antigen, which has been described for both NPCs [22,40] and MSCs [16,17,18,19].

The role of CD271 remains disputed, with different interpretations for various cell types. The most attention is devoted to its stem/progenitor characteristics. Pajanoja et al. suggested that the potency of neural crest stem cells (NCSCs) is intermediate between pluripotency and multipotency, as evidenced by the expression of pluripotency genes in the dorsal neural tube at the premigratory neural crest stage [41]. This explains their unique differentiation potential, which is not limited only to neurons and glial cells, but also extends to melanocytes and myofibroblasts [35,42,43]. Similarly, NCSC-derived keranocytes from elderly patients exhibited characteristics of neonatal cells according to cellular senescence prognostics [43]. Likewise, CD271+ population from MSCs exhibited better proliferation, clonogenicity and mesodermal differentiation abilities than CD271− cells [16,19,20], with higher expression of pluripotent genes [44]. CD271 has also been linked to a putative cancer stem cell population, as it has been found in glioma, glioblastoma, melanoma, and head and neck cancer cells [45,46,47,48,49,50], suggesting its potential oncogenic role [51].

The properties of CD271 are also being explored in other contexts, particularly with regard to pathological conditions. Its expression increases in adult neurons under pathological conditions such as cerebral ischemia, amyotrophic lateral sclerosis, nerve injury and Alzheimer’s disease [52]. Pathological conditions probably also alter the phenotype of CD271+ cells residing in mesenchymal stromal cells (MSCs), as these cells were found migrating to fractured bones in mice [53] or mobilized in peripheral blood of elder patients with myocardial infarction [54]. Filipp and colleagues observed dual effects observed in normal and pathological conditions, confirming that CD271 was associated with the proliferation and survival of melanoma-initiating cells as well as with the differentiation of normal melanocytes [55]. These interactions suggest that CD271 exerts a pleiotropic effect regulated by the local microenvironment. However, such relations have yet to be detailed for nervous tissue.

The association of CD271 with NCSCs residing within MSCs and its potential implications have not been fully explored in the scientific literature. The CD271+ population, isolated from CNS and PNS, was capable of self-renewal and differentiation into neurons and glial cells in vitro and in vivo [40,56]. In MSC studies, authors mostly confirmed the neural marker expression [44,57,58]. Derived exosomes enhanced axon regeneration, synapse formation and neovascularization in animal spinal cord injury (SCI) models, thereby improving regeneration [59,60]. The direct associations of MSC-CD271 with a neural phenotype remain to be explored.

### 3.2. Stem- and Neural-like Properties of the WJ-MSC-CD271+ Subpopulation

The main aim of the described studies was to characterize the CD271+ MSC subpopulation in the context of its neural differentiation potential. Wharton Jelly, a part of the umbilical cord, was chosen due to its abundant MSC population, characterized by highly expansive potential and non-invasive isolation procedure [27,28]. Although neonatal MSCs contain only a small proportion of CD271+ cells (less than 1%), these cells were found to be more stable than those in adult MSCs [16].

Based on our experience and the literature, we decided to isolate the CD271+ subpopulation by the fluorescence-activated cell sorting technique [61]. To obtain a sufficient number of cells for FACS separation, the WJ-MSCs were cultured up to the 3rd–5th passage, which is still considered an early passage. FACS enabled WJ-MSC-CD271+ subpopulation to be enriched from 1% up to 50%, providing 110 times more cells than in the initial population. However, technical concerns remained, as the sorting parameters indicated that not all positive cells in the initial population were detected and collected in the positive fraction. The negative population contained almost no CD271+ cells. Further flow cytometric analysis revealed a gradual decrease in the number of CD271+ cells in the positive population over the next two passages. The rapid loss of CD271+ cells in cell culture was also confirmed in the literature for MSCs derived from iPSC organoids [22]. Interestingly, the negative population also contained a proportion of cells, at a level similar to that observed in the unsorted population. Probably there could be some internal mechanism for CD271 renewal to maintain its expression at a constant level in the population.

The colony-forming unit (CFU) assay confirmed the higher clonogenic potential of CD271+ cells. WJ-MSC-CD271− formed significantly fewer colonies than WJ-MSC-CD271+. Other authors also confirmed our observations [16]. Despite their low abundance in the initial population, CD271+ cells constitute the proliferative fraction of the heterogeneous WJ-MSC population, indicating their stem/progenitor characteristics. The positive population contained more cells with stem characteristics, as confirmed by the CFU assay.

In further steps, we analyzed the potential neural phenotype of studied populations. WJ-MSC-CD271+ cells exhibited an increase in NESTIN gene expression immediately after FACS sorting. Nestin is not only an NPC marker, but is also characteristic for NCSCs isolated from adult tissues [36,38]. With further cell culture, we reported that NESTIN expression weakened and H3TUBULIN and MAP2 expression increased. Immunocytochemical staining revealed a significantly higher number of B-III-Tubulin-positive cells in the CD271+ population. A similar pattern occurs during the morphological and physiological changes in NSC/NPC neuronal differentiation. However, it should be emphasized that the expression of neuronal markers alone does not confirm neuronal identity or functional maturation. B-III-Tubulin is expressed during cell elongation and neurite outgrowth. Further maturation of neurons is accompanied by the replacement of β-III-Tubulin with MAP2 [62,63]. The authors reported comparable processes during the acquisition of an NSC-like phenotype by MSCs [5]. Alongside B-III-Tubulin, we reported elevated levels of A2B5—a ganglioside antigen typical for oligodendrocyte precursors, which also labels early neural progenitors [64].

In the next step, we introduced CD271+/CD271− cells to CSF or fragments of native tissue as OHC. Both of these components provide a variety of supporting factors like proteins, neurotransmitters or trophic factors mimicking the tissue microenvironment [65,66,67,68]. CSF is a natural component of the central nervous system and constitutes an important regulator of neural stem/progenitor cell proliferation, migration, survival, and differentiation during both embryonic and adult neurogenesis [68]. CSF is not only a nutritive fluid, but also an active component of the neural stem cell niche. Similarly, organotypic hippocampal slice cultures preserve intact cytoarchitecture, neuronal-glial interactions, extracellular matrix composition, and synaptic connectivity, thereby reproducing key aspects of the native neural environment [67,69,70,71]. Compared with simplified monoculture systems supplemented with selected recombinant factors, OHC-based models more faithfully reproduce physiological nervous tissue conditions while simultaneously allowing precise experimental control and reduction in in vivo animal experimentation. Under the implemented conditions, we also confirmed an increase in B-III-Tubulin and A2B5 at the protein level as well, suggesting CD271+ cells are drifting to a neuronal phenotype. We speculate that the delivered signals could impact the gene expression in the negative subpopulation. However, the microenvironment is insufficient to stimulate the positive population, requiring a stronger neural inductor. Taken together, these findings suggest that CSF and OHC conditions may primarily support or reveal a pre-existing neural-like bias in CD271-enriched cells rather than actively inducing neuronal differentiation.

### 3.3. Limitations and Further Perspectives

We have confirmed that WJ-MSC-CD271+ cells exhibit increased neuronal potential, which is a promising development for future applications. However, there are some issues that need to be addressed. Firstly, CD271 alone is insufficient to confirm the distinct neural crest-derived origin of the studied population. Currently, the WJ-MSC-CD271+ subpopulation is rather NSCS-like with enhanced neuronal phenotype. Nevertheless, Al-Bakri and colleagues have already confirmed that CD271+ cells isolated from umbilical cord blood co-expressed the other NCSCs markers [39]. Secondly, the observed MAP2 expression by CD271+ cells is very promising. However, to achieve a full neuronal maturation, more advanced protocols utilizing additional signaling molecules or 3D conditions should be applied [36,38]. Finally, the received low number of CD271+ and their further gradual loss during in vitro culture must also be addressed.

For this purpose, we chose to stimulate WJ-MSCs with EGF and bFGF—mitogens standardly used in NSCs culture [72]. A combination of EGF and bFGF is often applied for NPC expansion promotion. Depending on the EGF-bFGF cocktail concentration, NPCs differentiated into glutametric neurons (10 ng/mL) or cholinergic neurons (25 ng/mL) [73]. Similarly, they are added to induce MSCs neural differentiation [2,5,74,75], usually in combination with other signaling molecules such as retinoic acid, neurotrophins (NGF, GDNF, BDNF), B27 or N21, forskolin or sonic hedgehog [2,74]. The transcriptome of 3D cultured MSCs exhibited enrichment of genes associated with neurogenesis after EGF-bFGF stimulation [76]. bFGF treatment increased CD271 expression in NSCs derived from human ESCs, suggesting that the presence of neural crest precursors was regulated by extrinsic signals [14]. The EGF and bFGF strategy was proposed to improve the CD271 expression in dental-related stem cells [38], and to promote neuronal differentiation of CD271 isolated from umbilical cord blood in combination with BDNF [39]. In our study, EGF-bFGF stimulation not only increased CD271 expression in the initial population, but also allowed it to be preserved in subsequent in vitro culture. We observed the improvement of sorting efficacy as well. When EGF and bFGF were removed from the culture medium, CD271 expression dropped instantly, regardless of the previous stimulation. CD271, together with EGF and bFGF, is involved in MAPK/ERK and PI3/AKT signaling pathways, which regulate the proliferation, survival and differentiation of stem cells [77,78,79,80]. Simultaneous interaction promotes the preservation of early progenitor subsets like CD271+ within MSCS [81,82]. Our observation supports the hypothesis that CD271+ cells could constitute a pool of neural precursor cells within the heterogeneous MSC population, manifesting increased neuronal potential.

Although numerous studies demonstrated that MSCs could acquire neural-like characteristics under conditions involving exogenous neurogenic stimulation, including retinoic acid, growth factors, and commercially available neuronal differentiation media, such protocols often rely on strong artificial induction signals that may not fully reflect physiological cell behavior within the neural microenvironment [5,30,74,83]. However, the objective of the present study was different—to investigate whether a distinct subpopulation within the heterogeneous WJ-MSCs possesses an intrinsic predisposition to spontaneously respond to neural tissue-derived environmental cues rather than forcing neuronal differentiation through predefined neurogenic protocols. Therefore, we intentionally applied experimental conditions based on biologically relevant niche-related environments, including CSF and OHC to better mimic the complex signaling conditions present in vivo. This approach allowed us to assess spontaneous neural-oriented responses under physiologically relevant conditions rather than under forced differentiation paradigms. It remained unclear whether the CD271+ population would preferentially exhibit neuronal or glial differentiation tendencies at the stage of the experimental design. Consequently, we decided to incorporate a panel of specific markers into our analysis. The obtained results indicating a neuronal-oriented response, therefore provide an important basis for further studies focused on the biological role and therapeutic relevance of this MSC subpopulation.

## 4. Materials and Methods

### 4.1. WJ-MSCs Isolation and Culture

Human umbilical cords were acquired from the full-term deliveries with the written maternal consents. Tissue was transported in phosphate-buffered saline (PBS) (Sigma-Aldrich, Merck, Saint Louis, MO, USA) with an antibiotic-antimycotic mixture (penicillin, streptomycin, and amphotericin B, 1:100; Capricorn Scientific, Ebsdorfergrund, Germany). Then, umbilical cords were cut into slices with a lancet (2–3 mm in thickness). Wharton Jelly’s cylindrical fragments (diameter: 2–3 mm) were obtained with a biopsy punch (Miltex, GmbH, Viernheim, Germany) and transferred to adherent 6 well-plates with culture medium: DMEM (Gibco, Thermo Fischer Scientific, Walthman, MA, USA), 5% human platelet cell lysate PLTGold Clinical Grade (Mill Creek Life Sciences, Rochester, MN, USA) and an antibiotic-antimycotic mixture and cultured at 37 °C in 95% humidity, 5% CO_2_, and 5% O_2_. After cell migration from explants, WJ-MSCs were detached using the Accutase Cell Detachment Solution (Merck, Saint Louis, MO, USA), collected and counted. For the next 5 passages, WJ-MSCs were cultured in the described conditions and passaged when confluency reached around 80%.

### 4.2. Multipotent Differentiation Assay

WJ-MSCs’ multipotency was verified with mesodermal lineage differentiation assays. For this purpose, commercially available media were used for osteogenic, chondrogenic and adipogenic differentiation (Gibco, Thermo Fischer Scientific). Adipogenesis and chondrogenesis processes lasted 14 days, while osteogenesis—21 days. As the differentiation induction ended, the cells were fixed in 4% paraformaldehyde (PFA) and stained with histochemical dyes for further evaluation: 2% alizarin red S (for osteogenesis), 1% alcian blue (chondrogenesis) and 0.5% Oil Red O (adipogenesis).

### 4.3. Flow Cytometry

Flow cytometry was used for detection of the surface antigens recommended by ISCT. For this purpose, the Human MSC Analysis Kit (BD) was used with anti-human primary antibodies: CD73-APC, CD90-FITC, CD105-PerCP-Cy5.5 (positive cocktail), CD11b-PE, CD19-PE, CD34-PE, CD45-PE (negative cocktail) and respective isotype controls. CD271 expression was detected with the PE-CD271 antibody (BD), together with the respective isotype control (BD). For flow cytometry analysis, the cells were detached with the Accutase Cell Detachment Solution (BD) and washed in PBS. The required cell number (1 × 10^6^) was resuspended in cold Stain Buffer (BD) and then incubated with the antibodies in the dark for 30 min. After incubation, cells were washed twice with Stain Buffer (BD) and resuspended in Stain Buffer. Flow cytometry analysis was performed with the FACS Canto II (Becton Dickinson, Franklin Lakes, NJ, USA), while the results were evaluated using FACSDiva (version 6.1.3) and FlowJo 10 software (Becton Dickinson).

### 4.4. Fluorescence-Activated Cell Sorting (FACS)

WJ-MSCs from the 3rd–5th passages were used for the FACS separation of WJ-MSC-CD271+ and WJ-MSC-CD271− subpopulations. Cell staining was performed as described above, with the use of 10–20 × 10^6^ cells for the separation. WJ-MSCs were sorted using the FACS Aria IIu (BD) in the Laboratory of Cytometry, Nencki Institute of Experimental Biology, Warsaw. The gating strategy was presented in the online resources (Supplementary Figure S1). Both collected subpopulations were resuspended in PBS with the addition of 5% human platelet lysate and a 1:100 mixture of an antibiotic-antimycotic and then transported to our laboratory for further experiments.

The following FACS parameters were compared: recovery, yield, efficiency, and viability before and after sorting. Recovery was expressed as the ratio of the number of cells obtained in the positive fraction to the number of cells used in the sorting. To calculate yield, we compared the ratio of positive cell percentage before and after cell sorting. Efficiency was expressed as the ratio of the number of positive cells obtained in the positive fraction to the number of cells used in sorting. To estimate yield and efficiency, samples were analyzed with a flow cytometer. Viability was calculated by counting cells with Trypan Blue on a hemocytometer before and after the FACS separation.

### 4.5. Colony Forming Unit (CFU) Assay

CFU assay was performed to determine the percentage of stem cells in the population based on their ability to form colonies. Unsorted WJ-MSCs, WJ-MSC-CD271−, and WJ-MSC-CD271+ were seeded at a density of 10 cells per well in 6-well plates. After 10 days of culture, the cells were fixed with 4% PFA for 15 min, washed with PBS, and stained with 0.5% toluidine blue for 20 min. After staining, the cells were washed with distilled water. The number of colonies containing ≥50 cells was counted to calculate the percentage of CFU in the seeded cells.

### 4.6. Gene Expression Analysis

Total RNA was isolated from the following groups, depending on the experiment: unsorted WJ-MSCs; WJ-MSC-CD271− from the first and second passages after FACS; and WJ-MSC-CD271+ from the first and second passages after FACS. Total RNA was isolated using the Total RNA Mini Plus Concentrator Kit (A&A Biotechnology, Gdynia, Poland), following the manufacturer’s instructions. The quantity and quality of the RNA were then assessed using the NanoDrop 2000 spectrophotometer (Thermo Fisher Scientific). In case of genomic DNA contamination, RNA samples were washed with the Clean up RNA Concentrator (A&A Biotechnology).

Reverse transcription was performed using a High-Capacity RNA-to-cDNA™ Kit (Applied Biosystems) to generate complementary DNA (cDNA). Quantitative polymerase chain reaction (qPCR) was performed using SYBR Green Master Mix (Applied Biosystems) and specific primers (Table 2) with the 7500 Real Time PCR System (Applied Biosystems). The relative amount of RNA was calculated using the comparative delta-delta Ct method (2^−ΔΔCt^) and expression was normalized using the GAPDH gene. Depending on the experiment, samples from either unsorted WJ-MSCs or the negative population were chosen as the reference group. Gene expression was then compared to the mean level of corresponding gene expression in the reference group and expressed as an n-fold ratio.

### 4.7. Immunocytochemistry Staining

Immunocytochemical analysis was performed on the following groups, depending on the experiment: unsorted WJ-MSC, WJ-MSC-CD271+ and WJ-MSC-CD271−. For this purpose, the cells were seeded onto a 24-well plate containing poly-L-lysine-coated glass slides and cultured until they reached 80% confluence. Firstly, the cells were washed with PBS and fixed in 4% PFA for 15 min. The samples were then permeabilized with 0.2% Triton X-100 (Sigma-Aldrich) for 15 min and washed with PBS. Next, the samples were incubated with a blocking mix containing 10% goat serum (Sigma-Aldrich) for 1 h, after which the primary antibodies (Table 3) were applied and the samples were incubated at 4 °C for 24 h. The following day, the cells were washed with PBS and incubated with secondary antibodies conjugated with fluorochrome for 1 h. Cell nuclei were stained with mounting medium containing DAPI (Invitrogen, Thermo Fischer Scientific, Walthman, MA, USA). The samples were analyzed using the Zeiss LSM780 confocal microscope (Carl Zeiss, Oberkochen, Germany). Secondary antibody staining controls used for the experiments are presented in the online Supplementary Material (Figure S2).

### 4.8. Culture with Cerebrospinal Fluid (CSF)

CSF obtained from healthy patients was collected and centrifuged. The supernatant from 20 biological samples was pooled and cryopreserved for storage. The CSF samples were thawed directly before use.

WJ-MSC-CD271+ and WJ-MSC-CD271− cells were seeded at a density of 15,000 cells per cm^2^ under standard conditions. After 24 h, the culture medium was removed and the CSF was added. The CSF was replaced every three days, and the co-culture was conducted for seven days. After this time, the cells were collected for RNA isolation or fixed for immunocytochemical staining to examine neural differentiation processes.

### 4.9. Organotypic Hippocampal Slice Culture (OHC)

The hippocampi were obtained from 7-day-old Wistar rats after decapitation. Immediately after isolation, the hippocampi were cut into 400 µm slices using a McIlwain tissue chopper. Following PBS washing, the slices were transferred to semipermeable membranes (Millicell CM, Millipore Merck, Saint Louis, MO, USA) and placed in 6-well plates containing seeded WJ-MSC-CD271+ and WJ-MSC-CD271− cells. The cells and the hippocampi were cultured in DMEM (Gibco) containing 25% HBSS (Gibco), 5% human platelet lysate (PLTGold Clinical Grade, Mill Creek Life Sciences), 1 M HEPES (Gibco), 5 mg/mL glucose (Sigma-Aldrich) and an antibiotic-antimycotic solution (Genos). After 7 days of the coculture, the cells were collected for RNA isolation or fixed for immunocytochemical staining to examine the neural differentiation processes.

### 4.10. Cell Culture in the Presence of EGF and bFGF

The change in the content of CD271+ cells in WJ-MSC populations was compared after the addition of growth factors: 20 ng/mL of both EGF and bFGF (Gibco) were added to the standard medium culture. The growth factors were added one passage before fluorescence-activated cell sorting (FACS) and for the next two passages after separation. The CD271+ cell content was then compared using flow cytometry.

### 4.11. Statistical Analysis

The following groups were analyzed depending on the experiment: unsorted WJ-MSC, WJ-MSC-CD271− and WJ-MSC-CD271+. For each experiment, cells were obtained from three WJ donors, and a mean value was calculated based on three technical repetitions. The Shapiro–Wilk normality test was applied. Data from two-group comparisons were analyzed using the Student’s *t*-test, while data from multiple groups were analyzed using one-way analysis of variance (ANOVA) and Tukey’s multiple comparisons test as a post-hoc. For non-normal distributions, the Mann–Whitney test was used for two-group comparisons and the Kruskal–Wallis test for multiple groups. All results were presented as the mean ± standard deviation (SD) and were considered statistically significant when *p* < 0.05. Statistical analysis of the data was performed using GraphPad Prism 7.

## 5. Conclusions

In summary, our observations confirmed the distinctive characteristics of the CD271+ subpopulation derived from WJ-MSCs. Although CD271 alone is not sufficient to confirm the neural crest character of this subpopulation, we observed increased expression of neuronal genes and proteins, similar to the patterns seen during NPC differentiation to neurons. These patterns were also observed during contact with CSF, an important component of nervous tissue, and in the presence of native tissue during OHC. Together with the increased clonogenic potential observed, these findings suggest that the CD271+ subpopulation exhibits NPC characteristics. However, due to the small size of the CD271+ population within the WJ-MSCs, treatment with EGF and bFGF is crucial to maintain the CD271+ population in subsequent in vitro cultures. Furthermore, the conclusions of this study are primarily based on analyses of marker expressions, which do not confirm functional neuronal differentiation, such as electrophysiological activity or synaptic integration. Further studies are therefore required to determine whether these cells acquire functional neuronal properties.

## Figures and Tables

**Table 1:** Parameters defined for CD271+ separation.

Sorting Parameter	Mean	SD
CD271+ cells recovery, %	22	19.05
Yield	110	39
Sorting efficiency, %	18.38	5.18
Cell viability before FACS, %	95	5.22
Cell viability after FACS, %	95.21	2.66

**Table 2:** List of analyzed genes for RT-qPCR.

Gene	NCBI Reference Sequence	Product Size (bp)	Primer Sequence (5′ -> 3′)
GAPDH	NM_001357943.2	141	F: GACGCTGGGGCTGGCATTG
			R: GCTGGTGGTCCAGGGGTC
NESTIN	NM_006617.2	64	F: GGGAAGAGGTGATGGAACCA
			R: AAGCCCTGAACCCTCTTTGC
H3TUBULIN	NM_001197181.2	126	F: GGAAGAGGGCGAGATGTACG
			R: GGGTTTAGACACTGCTGGCT
MAP2	NM_001375545.1	99	F: TTGGTGCCGAGTGAGAAGA
			R: GTCTGGCAGTGGTTGGTTAA
GFAP	NM_001363846.2	100	F: CCGACAGCAGGTCCATGT
			R: GTTGCTGGACGCCATTG
S-100β	NM_006272.3	91	F: AGCGCTCCTGGAAAAAGCAA
			R: TTGAATCGCATGGGTCACGG
NG2	NM_001897.5	118	F: GTCTACACCATCGAGCAGCC
			R:TGTGTGAGAACAGCACGAGC

**Table 3:** List of analyzed proteins for immunocytochemistry.

Antigen	Isotype	Dilution	Company	Catalogue Number	Applied Secondary Antibody	Secondary Antibody Fluorochrome
Nestin	Mouse IgG1	1:500	Merck Millipore	MAB5326	Goat anti-IgG1	Alexa Fluor 546
β-III-Tubulin	Mouse IgG2B	1:500	Sigma-Aldrich	T8660	Goat anti-IgG2B	Alexa Fluor 546
MAP2	Mouse IgG1	1:1000	Merck Millipore	M4403	Goat anti-IgG1	Alexa Fluor 546
GFAP	Rabbit IgG H + L	1:500	Dako (Agilent Technologies, Glostrup, Denmark)	Z0334	Goat anti-IgG H + L	Alexa Fluor 488
S-100β	Rabbit IgG H + L	1:200	Abcam (Cambridge, United Kingdom)	AB52642	Goat anti-IgG H + L	Alexa Fluor 488
A2B5	Mouse IgM	1:400	Merck Millipore	MAB312	Goat anti-IgGM	Alexa Fluor 546

## Data Availability

The original data presented in the study are openly available in the Repository for Open Data RepOD at https://doi.org/10.18150/D7EMSF (accessed: 22 May 2026).

## Supplementary Materials

The following supporting information can be downloaded at: https://www.mdpi.com/article/10.3390/ijms27114896/s1.

## Abbreviations

The following abbreviations are used in this manuscript:

bFGF	Basal Fibroblast Growth Factor
CFU	Colony-Forming Unit
CNS	Central Nervous System
CSF	Cerebrospinal Fluid
EGF	Epidermal Growth Factor
ESCs	Embryonic Stem Cells
FACS	Fluorescence-Activated Cell Sorting
iPSCs	Induced Pluripotent Stem Cells
ISCT	International Society for Cell and Gene Therapy
MSCs	Mesenchymal Stem/Stromal Cells
NC	Neural Crest
NCSCs	Neural Crest-Derived Stem Cells
NPCs	Neural Progenitor Cells
NSCs	Neural Stem Cells
OHC	Organotypic Hippocampal Slices Culture
PBS	Phosphate Buffer Saline
PFA	Paraformaldehyde
PNS	Peripheral Nervous System
SCI	Spinal Cord Injury
SCs	Stem Cells
WJ	Wharton Jelly
