# Supplementary material for: CD271 Identifies a Subpopulation with Enhanced Neural-like Potential Within Wharton Jelly Derived Mesenchymal Stem/Stromal Cells
Source: Int J Mol Sci. 2026 May 28;27(11):4896. doi: 10.3390/ijms27114896 (PMC13257159; doi:10.3390/ijms27114896)
Supplement: Supplementary file 1 [file ijms-27-04896-s001.zip › ijms-4309918-supplementary.pdf]

Table S1. CD271+ sorting parameters after EGF and bFGF stimulation

Sorting parameter	Mean	Standard deviation
CD271+ cells recovery, %	68.9	32.7
Yield	59.5	38.7
Sorting efficiency, %	36.6	21.7

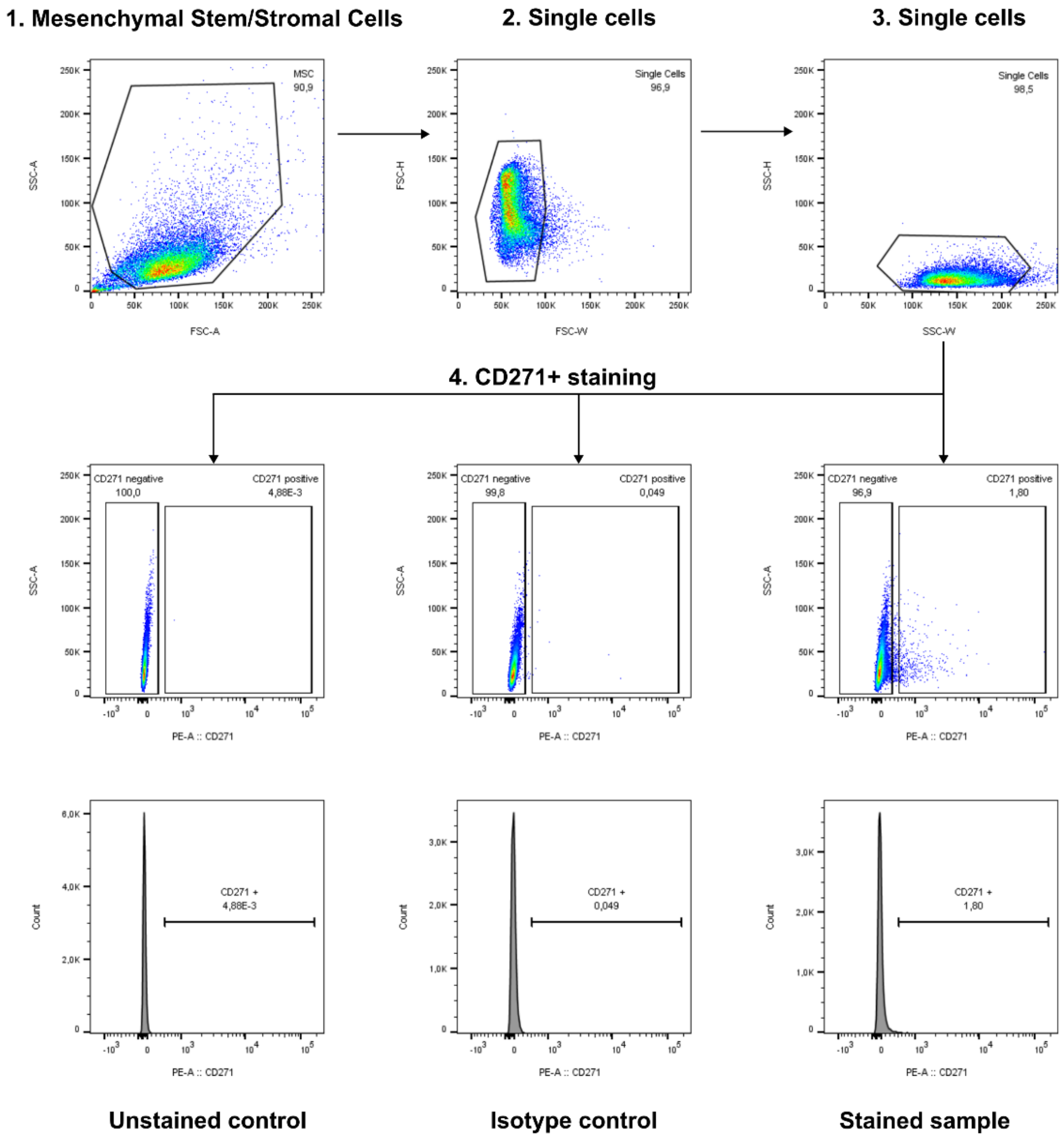

Figure S1. Strategy gating for sorting of SSEA-4+ cells in WJ-MSCs for unstained control, isotype control and stained sample; flow cytometry, FACSAria II.

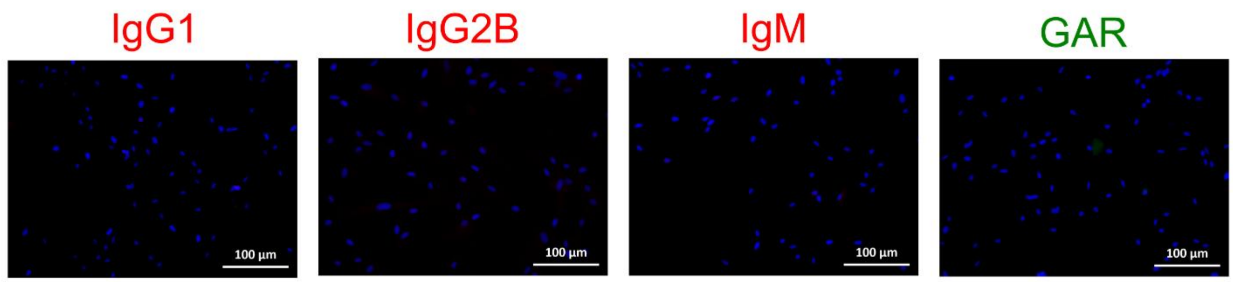

Figure S2. Secondary antibody staining controls used for the experiments. Following secondary antibodies were applied: goat anti-mouse IgG1-Alexa Fluor 546, goat anti-mouse IgG2B-Alexa Fluor 546, goat anti-mouse IgM-Alexa Fluor 546 and goat anti-rabbit IgG H+L-Alexa Fluor 488 (GAR). Cell nuclei were stained with DAPI. Scale bars: 100 µm
